# *Pixel* calculations using *Orca* or *GAUSSIAN* for electron density automated within the *Oscail* package

**DOI:** 10.1107/S1600576721008529

**Published:** 2021-09-29

**Authors:** Patrick McArdle

**Affiliations:** a School of Chemistry, National University of Ireland, Galway, University Road, Galway, H91 TK33, Ireland

**Keywords:** *Pixel*, lattice energy, *Orca*, *GAUSSIAN*

## Abstract

Many discussions of the intermolecular interactions in crystal structures concentrate almost exclusively on an analysis of hydrogen bonding. A simple analysis of atom–atom distances is all that is required to detect and analyse hydrogen bonding. However, for typical small-molecule organic crystal structures, hydrogen-bonding interactions are often responsible for less than 50% of the crystal lattice energy. It is more difficult to analyse intermolecular interactions based on van der Waals interactions. The *Pixel* program can calculate and partition intermolecular energies into Coulombic, polarization, dispersion and repulsion energies, and help put crystal structure discussions onto a rational basis. This Windows PC implementation of *Pixel* within the *Oscail* package requires minimal setup and can automatically use *GAUSSIAN* or *Orca* for the calculation of electron density.

## Abstract 2

The *Pixel* program can be run automatically by the *Oscail* package using either *GAUSSIAN* or *Orca* to calculate electron density.

## 1. Introduction   

An understanding of the intermolecular interactions in crystal structures can help in the design of active pharmaceutical ingredient derivatives or coformers for cocrystals (MacLeod & Muller, 2012 ▸; Chen & Trout, 2010 ▸; Karimi-Jafari *et al.*, 2018 ▸). Many crystal structure discussions are limited to discussions of hydrogen-bonding interactions which are readily detected by an analysis of atom–atom distances. However, the fractional contribution of hydrogen bonding to the lattice energy can vary over a wide range. For example, in aspirin [Cambridge Structural Database (CSD; Groom *et al.*, 2016 ▸) code ACSALA27; Tyler *et al.*, 2020 ▸], hydrogen bonding accounts for 66% of the lattice energy, compared with only 22% of the lattice energy of naproxen (CSD code COYRUD14; Hachuła, 2018 ▸). Concentrating on hydrogen bonding will often ignore important van der Waals interactions, which may lead to a poor understanding of the origin of important material properties. The computer programs *Pixel* (Gavezzotti, 2005 ▸) and *CrystalExplorer* (Mackenzie *et al.*, 2017 ▸) can be used to calculate lattice energies and obtain an estimate of the intermolecular energies, partitioned into Coulombic, polarization, dispersion and repulsion components. This information can form the basis of a more realistic analysis of crystal structure than an examination of hydrogen bonding alone.

The *Pixel* program as provided by the author (Gavezzotti, 2005 ▸) consisted of Fortran source files, batch files and data files designed for use at the DOS prompt on a Windows PC. The Windows-PC-based *Oscail* software package provided automation of the *Pixel* procedures (McArdle, 2017 ▸) and *MrPIXEL* (Reeves *et al.*, 2020 ▸), which operates within *Mercury* which is part of the CSD software, also automates *Pixel* operations. The *CrystalExplorer* software, which can provide a similar analysis, was developed for use with Mac and Linux systems and is also available in a Windows version. The Mac and Linux versions of *CrystalExplorer* can use *GAUSSIAN* (Frisch *et al.*, 2016 ▸) or the *Tonto* quantum chemistry package (Jayatilaka & Grimwood, 2003 ▸) for electron-density calculation. *Tonto* is available on an academic free basis. The Windows-PC version of *CrystalExplorer* can use *GAUSSIAN* or *Tonto*. Using *Tonto* on a Windows PC requires cross-compilation of the code on a Linux system. The version of the *Oscail* software package described here has a much improved *Pixel* interface and can use *GAUSSIAN* or the *Orca* quantum chemistry program (Neese, 2018 ▸) combined with *MultiWFN* (Lu & Chen, 2012 ▸) for electron-density calculation and thus provides access to realistic crystal structure analysis using software that requires minimal setup, runs in parallel mode and is available on an academic free basis.

## 2. Overview of running *Pixel* using *Oscail*   

**Figure 1:**
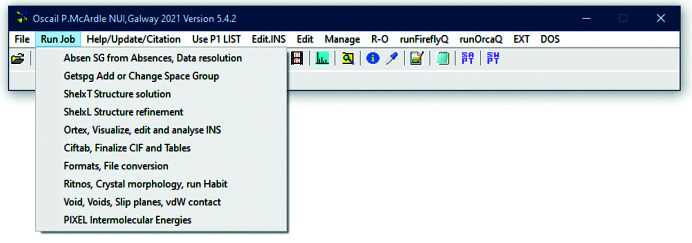
Starting *Pixel* within *Oscail*.

**Figure 3:**
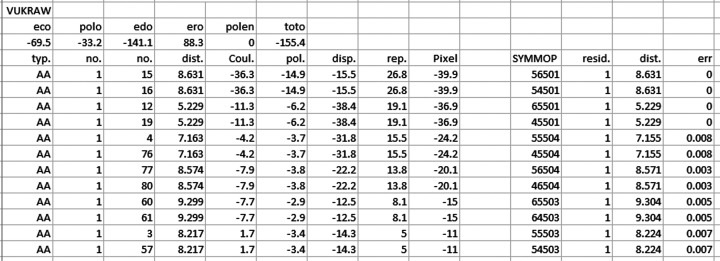
*PixelC* output for CSD refcode VUKRAW.

Starting *Pixel* within *Oscail* and the options available are shown in Figs. 1 ▸ and 2 ▸, respectively. Starting from a suitable CIF, *Oscail* can run *GAUSSIAN* or *Orca* to generate electron-density files and then automatically run *PixelC*. Molecule centroid geometry information generated by *Oscail* can then be used to generate symmetry codes for the molecule pairs in the .MLP file written by *PixelC*. *PixelC* and *Oscail* both use mass-weighted centroids. The condensation level is the number of points in the electron-density cube that are merged to give one pixel. The symmetry codes can be automatically added to the .MLP file in a format suitable for spreadsheet use (Fig. 3 ▸). This example, 4-hydroxy-*N*-phenylbenzenesulfonamide (CSD refcode VUKRAW; Walshe *et al.*, 2015 ▸), is provided with *Oscail*. The quantity labelled ‘toto’ is the lattice energy. In the main part of the table the quantities dist., Coul., pol., disp., rep. and Pixel are the centroid distances and the Coulombic, polarization, dispersion, repulsion and total energies for the interaction, respectively. The errors given in the last column are usually very small and indicate that the program has found a match between the *Oscail* and *PixelC* centroid distances. If there is a large error then the default 20 Å limit on the *Oscail* centroid scan should be increased. More details are available in the original *Pixel* documentation which is supplied with *Oscail*.

**Figure 4:**
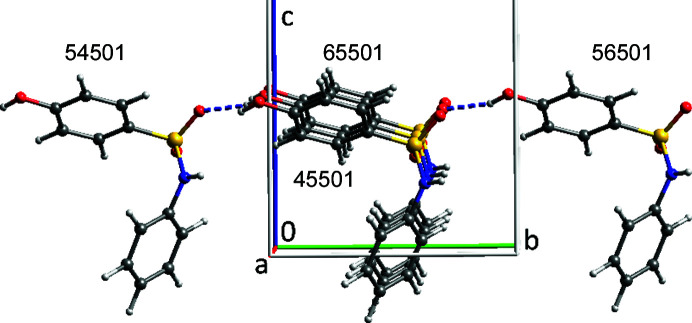
The four strongest interactions added to the asymmetric unit of VUKRAW.

The *ORTEP*-type symmetry codes (Burnett & Johnson, 2000 ▸) allow straightforward on-screen visualization of the important intermolecular interactions in the VUKRAW structure (Fig. 4 ▸). The two strongest interactions with large Coulombic contributions are the hydrogen-bonding inter­actions, and the two −36.9 kJ mol^−1^ interactions are dominated by dispersion. It is the latter interactions in which the molecules are efficiently stacked that drive the needle growth observed in this system (Walshe *et al.*, 2015 ▸). *ORTEP* symmetry codes are the translations along *x*, *y* and *z* + 5 and the space-group symmetry operation number. Thus, 55501 represents no translation on *x*, *y* or *z* and the symmetry operation number 1. The symmetry operation number may not be the same as that in the CIF. *Oscail* provides a translation of *ORTEP* symmetry codes into standard form (Table 1 ▸).

### 2.1. Important details   

The *Pixel* programs were designed to use CIFs downloaded from the CSD, and CIFs from other sources may cause problems. *Oscail* provides a routine for converting CIFs generated by *SHELXL* (Sheldrick, 2015 ▸) to CSD format, and this may work with CIFs from other sources. Running the *CLPCRY* program is a simple way to test a CIF. If there are problems, manual editing of the CIF to bring it closer to the format of a CSD CIF may be required.

It is also important to use H-atom positions close to those expected from neutron diffraction, rather than positions from X-ray diffraction. The *Pixel* input routines can adjust H-atom positions from X-ray diffraction data to neutron diffraction values, but we have found that the *ORTEX* program within *Oscail* is more satisfactory for making this adjustment. If the structure was determined by neutron diffraction or if it contains no H atoms then the program’s warnings about the use of neutron diffraction H-atom positions should be ignored.

*Oscail* generates input for both *GAUSSIAN* and *Orca* and, when the user is offered the choice to run *Orca* or *GAUSSIAN*, if the choice is not to run either program then manual intervention is possible. This type of intervention is necessary if, for example, the spin multiplicity needs to be changed from the singlet default value. The input files, .ino for *Orca* and .gjf for *GAUSSIAN*, can be edited using a text editor. It is not a good idea to edit the *GAUSSIAN* .gjf input files with *Gaussview* as *Gaussview* can only be used with basic .gjf files. The DOS .bat file jobname_o.bat can then be used to run *Orca*.

## 3. Calculating electron density using *Orca*   

*Pixel* writes input files for *GAUSSIAN* which work without problems. However, getting electron density from the *Orca* quantum chemistry package to work with *Pixel* was not straightforward. Density files written by *Orca* are all-electron-density files, rather than the valence-electron-density files which are written by *GAUSSIAN*. There is a switch within *PixelC* which allows the program to use all-electron-density files. Attempts to use this option with *Orca* density files were not satisfactory. The results were in some cases close to those obtained using *GAUSSIAN* electron-density files, but in most cases they made little sense and were dependent on the condensation level.

A satisfactory solution to the problem is to use *Orca* to calculate the wavefunction and export it in a suitable format that can be read by *MultiWFN* (Lu & Chen, 2012 ▸). *MultiWFN* is a wavefunction analysis program that can modify a wavefunction and perform a range of calculations on wavefunctions. *Oscail* automatically generates the *Orca* input, runs the *Orca* wavefunction calculation and sends the wavefunction to *MultiWFN*, where it is modified to have a frozen core and then used to calculate electron-density files. The *PixelC* results from these density files are within 0.2 kJ mol^−1^ of those obtained from *GAUSSIAN* electron density for calculations on uncharged systems.

## 4. Modifications made to *PixelC*   

The version of *PixelC* included in the current version of *Oscail* has been improved relative to the previous version (McArdle, 2017 ▸). The most important changes are that the 100-atom limit has been raised to 200 atoms, compilation of the program has been optimized and a version which is limited to calculation of intermolecular energies has been added (see Section 7). The optimized and intermolecular energy versions are, respectively, 2.2 and 6.7 times faster running CSD refcode VUKRAW than the default compilation. The main output file is now user friendly and spreadsheet compatible (Fig. 3 ▸).

## 5. Limitations of *PixelC*   

*PixelC* can only be used with complete molecules and it is limited to two molecules in the asymmetric unit. Complete molecules can in most cases be generated by using a suitable symmetry operation and the space-group symmetry can then be reduced. This procedure will often require a change of space-group origin. It has been suggested that, when making this change, it is better to use a non-standard setting of the space group in preference to a change of origin (Reeves *et al.*, 2020 ▸). β-Phthalocyanine (CSD refcode PHTHCY14; Jiang *et al.*, 2018 ▸) has half molecules on inversion centres in space group *P*2_1_/*n*, and the procedure for completing the molecules and changing to space group *P*2_1_ with a change of origin is described in a tutorial supplied with *Oscail*. A procedure for calculating pairwise intermolecular energies for structures with more than two molecules in the asymmetric unit is described below.

## 6. Indicative structure analysis   

**Figure 5:**
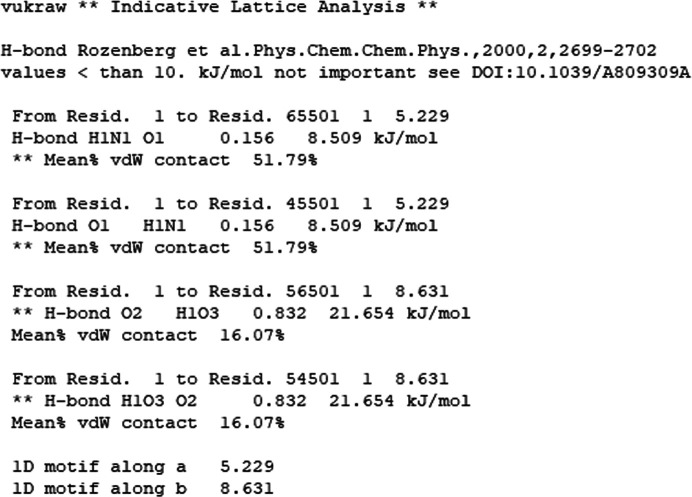
The strongest interactions in the indicative structure analysis of the VUKRAW structure.

Using a molecule centroid search for contacts, with a distance limit normally set to 20 Å, *Oscail* can generate a list of significant intermolecular contacts in which intermolecular atom–atom distances inside the van der Waals radii are used to indicate hydrogen-bond formation and intermolecular van der Waals contact fractions are used to indicate dispersion energies. The hydrogen-bond energies are estimated using a simple exponential function based on vibrational analysis (Rozenberg *et al.*, 2000 ▸) and the van der Waals contact fractions listed are defined as the fraction of the atoms in a molecule that are closer than the sum of the van der Waals radii + 1 Å to atoms in another molecule. These atom fractions have been shown to correlate with dispersion energies (Walshe *et al.*, 2015 ▸). The presence of 1D motifs in hydrogen bonding or van der Waals contact stacking is also detected. If the intermolecular energies involved are dominant among the contributions to the lattice energy, 1D motifs can be important drivers of anisotropic crystal growth (Walshe *et al.*, 2015 ▸). The strongest hydrogen bonds (H-bond) and the highest van der Waals (vdW) contact fractions in the list produced for VUKRAW are shown in Fig. 5 ▸, together with an indication of a van der Waals contact stack along **a** and a hydrogen-bond chain along **b**. A warning about weak hydrogen bonds is also given. The interactions in Fig. 5 ▸ correspond to the highest intermolecular energies calculated by *PixelC* (see Fig. 3 ▸). This indicative analysis can be used to guide a series of pairwise intermolecular energy calculations or to drive an automated calculation of the most important intermolecular energies for systems with *Z*′ values greater than 2. The automated use of the VUKRAW indicative structure analysis to drive *PixelS* (see Section 7) gave a lattice energy estimate of −154.5 kJ mol^−1^, which is close to the *PixelC* value of −155.4 kJ mol^−1^ (‘toto’ in Fig. 3 ▸). The indicative structure analysis is best done with full molecules but it does not require the change of space group described for *PixelC* calculation on PHTHCY14 in Section 5.

## 7. Structures with *Z*′ greater than 2   

**Figure 6:**
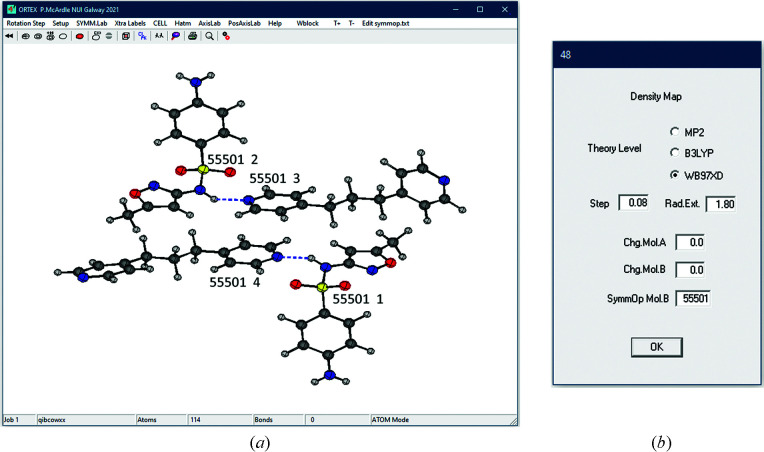
(*a*) The QIBCOW structure shown with symmetry codes and residue numbers, and (*b*) the *PixelS* dialogue.

The intermolecular energies in structures which have *Z*′ greater than 2 can be calculated in a pair-wise fashion using *PixelS*, a modified version of *PixelC* first added to *Oscail* in an update in 2018 (McArdle, 2021 ▸). The molecular pairs and the symmetry operations required to examine the most important intermolecular interactions are provided by the indicative estimate of intermolecular energies described above. The example used in the tutorials supplied with *Oscail* is 4-amino-*N*-(5-methyl-1,2-oxazol-3-yl)benzene-1-sulfonamide-4,4′-(propane-1,3-diyl)dipyridine (CSD refcode QIBCOW; Alsubaie *et al.*, 2018 ▸), which has a *Z*′ of 4 [Fig. 6 ▸(*a*)]. The molecule pairs are selected on screen and the symmetry operation required for the second molecule is added in the *PixelS* dialogue [Fig. 6 ▸(*b*)].

## 8. Comparison with energies calculated by *CrystalExplorer* and *Pixel*   

The strongest intermolecular interactions in the 1:1 pyridine–formic acid co-crystal (CSD refcode QAFFIM; Wiechert & Mootz, 1999 ▸) were calculated using *CrystalExplorer* to be −53, −13 and −7 kJ mol^−1^ (Mackenzie *et al.*, 2017 ▸). Using *PixelC*, both *GAUSSIAN* and *Orca*/*MultiWFN* gave −51, −13 and −9 kJ mol^−1^ for the same interactions. The *GAUSSIAN* and *Orca* calculations used the program defaults, which are 6-31G** basis sets and the density functional theory (DFT) functionals WB97XD and WB97X-D3, respectively, and *CrystalExplorer* employed the same basis sets and CE-B3LYP DFT functionals. *CrystalExplorer* results are also available for a salt cocrystal of pyridine and formic acid with the formula [C_5_H_6_N]^+^[HCO_2_]^−^·(HCO_2_H)_3_ (CSD refcode QAFFOS; Wiechert & Mootz, 1999 ▸). The two strongest anion–cation interactions and the strongest anion–anion and cation–cation repulsions reported were −253, −199, 342 and 268 kJ mol^−1^, respectively. Using the indicative estimate of structure inter­actions, *Oscail* can drive *PixelS* automatically to calculate the most significant interactions. The results obtained for the interactions above were −274, −243, 322 and 249 kJ mol^−1^, respectively, using *Orca*/*MultiWFN* electron density, and −285, −239, 322 and 248 kJ mol^−1^, respectively, using *GAUSSIAN* electron density. While undoubtedly the presence of charged species amplifies the differences in the calculations, the results are in reasonable agreement. A second polymorph of this salt cocrystal has been crystallized from a melt under high pressure (CSD refcode QAFFOS01; Lee *et al.*, 2016 ▸). These two polymorphs have different space groups but their structures are related. The energies of the interactions corresponding to those given above calculated by *PixelS* for QAFFOS01 using *Orca*/*MultiWFN* electron density were slightly higher, −294, −301, 320 and 272 kJ mol^−1^, respectively, as might be expected at higher pressure. An interesting difference between the polymorphs is that the anion in QAFFOS which was crystallized from solution made its three strongest interactions to formic acid molecules with those in the asymmetric unit. In QAFFOS01 two of the three strongest interactions made by the anion with formic acid molecules were outside the asymmetric unit. It is possible that the structure of QAFFOS preserves a molecular cluster from solution that might have been involved in nucleation and/or proton transfer (Anderson & Steed, 2007 ▸; Steed & Steed, 2015 ▸).

When the asymmetric unit of a crystal structure contains less than a full molecule, *PixelC* calculations require a completed molecule and a reduction in space-group symmetry as described in Section 5. While *PixelS* also requires a completed molecule it does not require a space-group change. This simplification arises for two reasons: firstly, when the input files for *PixelS* are being generated by the *ORTEX* program, within *Oscail*, additional coincident atoms are automatically dropped, and secondly, because *PixelS* does not use space-group symmetry operations. Using a completed molecule of Cr(CO)_6_ (CSD refcode FOHCOU02; Rees & Mitschler, 1976 ▸) and space group *Pca*2_1_, an indicative structure-analysis-driven *PixelS* calculation gave results in good agreement with published values (Table 2 ▸). To obtain the results given in Table 2 ▸ the α(O) value was increased from 0.75 to 1.0, as previously suggested for a coordinated CO ligand (Maloney *et al.*, 2015 ▸).

## 9. Examples without hydrogen bonding   

**Figure 7:**
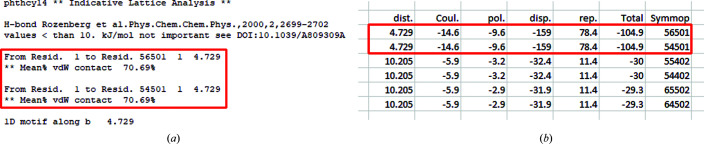
(*a*) Indicative analysis of PHTHCY14. (*b*) PHTHCY14 *PixelC* intermolecular energies.

β-Phthalocyanine (CSD code PHTHCY14) is not expected to have any significant hydrogen bonding and the indicative structure analysis and *PixelC* both suggest the presence of a dominant 1D motif along the *b* axis. This motif is based on dispersion energy and is responsible for the needle growth observed for crystals of β-phthalocyanine (Fig. 7 ▸) (Walshe *et al.*, 2015 ▸).

**Figure 8:**
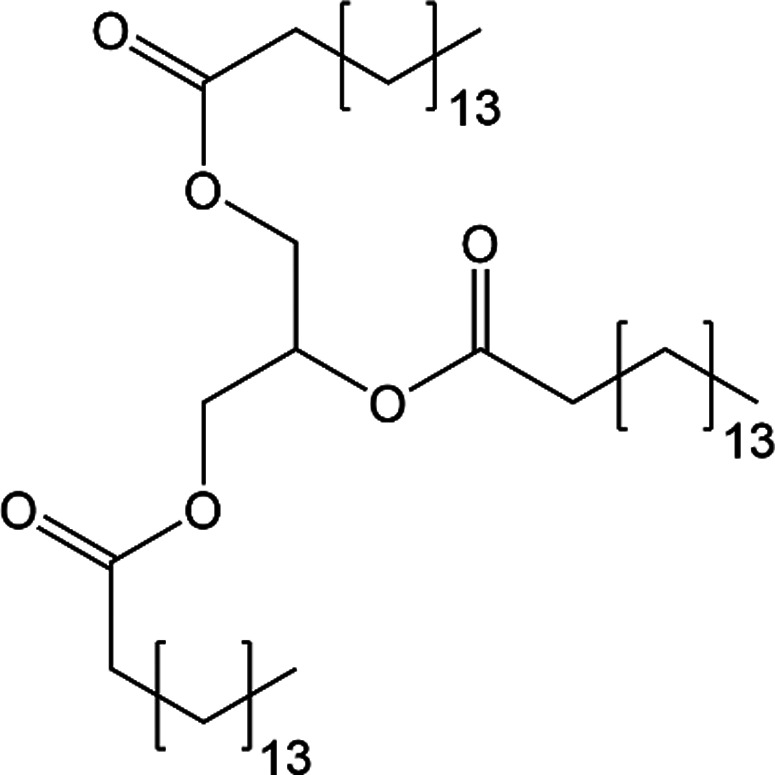
Structural formula of tripalmitin.

Tripalmitin (CSD refcode SUWMAY; van Langevelde *et al.*, 1999 ▸) is a triglyceride of palmitic acid (Fig. 8 ▸). It also grows as needles and is not expected to have significant hydrogen bonding in its structure. The formula has 155 atoms and is a test of the 200-atom version of *PixelC*. In this case there is no dispersion-dominated 1D motif in the observed needle growth direction along the *a* axis and needle growth is driven by other mechanisms (Boerrigter *et al.*, 2002 ▸).

## 10. Calculated and experimental lattice energies   

Lattice energies calculated for organic compounds using *Pixel* (Chickos & Gavezzotti, 2019 ▸) and DFT calculations with periodic boundary conditions (Marchese Robinson *et al.*, 2019 ▸) have been compared with experimental results. In these papers it was concluded that *Pixel* had a 68% chance of matching the experimental sublimation enthalpy with a root-mean-square deviation of 5.5 kJ mol^−1^ and that DFT could match experimental results with a root-mean-square error of 37 kJ mol^−1^. It is difficult to make any definite statement as to which is the more accurate approach. The correction of experimental results using a −2RT correction to allow for the neglect of vibrational motions of the crystal structure and differences between vibrations of the molecules in the gas and solid phases is favoured by some authors (Marchese Robinson *et al.*, 2019 ▸), while others believe that no correction is better than a bad correction (Chickos & Gavezzotti, 2019 ▸). There is no doubt that currently very expensive DFT calculations which take account of vibrational motion can yield highly accurate results which may be capable of reproducing the absolute energies of and energy differences between polymorphs (Fowles *et al.*, 2021 ▸). Calculations which take account of vibrational energy may in the future be the method of choice for lattice energy calculation.

## 11. Program availability and installation   

*Oscail* can be installed under most versions of MS Windows and is available in 32- and 64-bit versions. The installation includes all of the *Pixel* files. The software may be downloaded from http://www.nuigalway.ie/cryst on an academic free basis. A video and tutorials are also available. Commercial users must obtain permission for its use. Instructions for downloading and installing *Oscail* and software external to *Oscail* are provided in the Help files and on the Download web page.

## Figures and Tables

**Figure 2:**
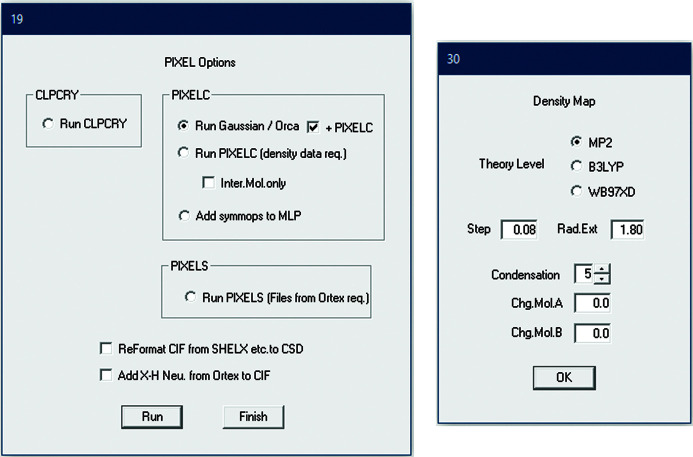
*Pixel* options dialogues.

**Table 1:** Energy components (kJ mol^−1^) for the interactions between molecules in the SUWMAY structure

Distance (Å)	Coulomb	Polarization	Dispersion	Repulsion	Total	Symmetry operation
7.383	−23	−14	−163.5	84	−116.5	1-x, 1-y, 2-z
5.451	−31.9	−13.4	−133.1	64.3	−114	1+x, y, z
5.451	−31.9	−13.4	−133.1	64.3	−114	-1+x, y, z
9.97	−38.3	−14.5	−112.8	57.7	−107.9	1-x, -y, 2-z
11.531	−17.3	−9.2	−137.2	68.7	−95	-x, -y, 2-z
8.058	−21.8	−8	−94.9	42.6	−82.2	-x, 1-y, 2-z

**Table 2:** Energy components (kJ mol^−1^) for the interactions between molecules in the Cr(CO)_6_ structure All calculations used 6-31G** basis sets.

Cr⋯Cr (Å)	DFT	Coulomb	Polarization	Dispersion	Repulsion	Total	Reference
6.203	CE-B3LYP	−5.8	−0.4	−13.9	8.3	−11.7	Mackenzie *et al.* (2017 ▸)
	WB97X-D3	−5.9	−1.7	−15.5	12	−11.1	This work
6.244	CE-B3LYP	−6	−0.4	−13.9	8.8	−11.5	Mackenzie *et al.* (2017 ▸)
	B3LYP	−5.8	−1.8	−18.7	13	−13.3	Maloney *et al.* (2015 ▸)
	WB97X-D3	−8.4	−2.8	−15.4	12.5	−14.2	This work
6.882	CE-B3LYP	−4.4	−0.3	−8.4	5	−8	Mackenzie *et al.* (2017 ▸)
	B3LYP	−4.3	−1.1	−11.2	7.4	−9.1	Maloney *et al.* (2015 ▸)
	WB97X-D3	−4.4	−1.4	−9.2	7.4	−7.6	This work
